# Effects of Untreated Drinking Water at Three Indigenous Yaqui Towns in Mexico: Insights from a Murine Model

**DOI:** 10.3390/ijerph18020805

**Published:** 2021-01-19

**Authors:** Sofia Navarro-Espinoza, Aracely Angulo-Molina, Diana Meza-Figueroa, Guillermo López-Cervantes, Mercedes Meza-Montenegro, Aurora Armienta, Diego Soto-Puebla, Erika Silva-Campa, Alexel Burgara-Estrella, Osiris Álvarez-Bajo, Martín Pedroza-Montero

**Affiliations:** 1 Department of Geology, University of Sonora, Rosales and Encinas, Hermosillo 83000, Sonora, Mexico; sofia.navarro@unison.mx; 2 Department of Biological Chemical Sciences, University of Sonora, Rosales and Encinas, Hermosillo 83000, Sonora, Mexico; aracely.angulo@unison.mx; 3 Department of Physics Research, University of Sonora, Rosales and Encinas, Hermosillo 83000, Sonora, Mexico; diego.soto@unison.mx (D.S.-P.); erika.silva@unison.mx (E.S.-C.); alexel.burgara@unison.mx (A.B.-E.); osiris.alvarez@unison.mx (O.Á.-B.); 4 Department of Medicine, University of Sonora, Rosales and Encinas, Hermosillo 83000, Sonora, Mexico; guillermo.lopez@unison.mx; 5 Department of Natural Resources, Sonora Technological Institute, 5 de Febrero 818 Sur, Obregon City 85000, Sonora, Mexico; mmeza@itson.edu.mx; 6 Institute of Geophysics, National Autonomous University of Mexico-UNAM, Coyoacán 04510, Ciudad de Mexico, Mexico; victoria@geofisica.unam.mx; 7 Consejo Nacional de Ciencia y Tecnología CONACyT, Insurgentes 1582, Benito Juárez 03940, Ciudad de Mexico, Mexico

**Keywords:** indigenous towns, drinking water, murine model, arsenic, manganese

## Abstract

Background: Reports in a northwestern Mexico state linked arsenic (As) in drinking water to DNA damage in people from indigenous communities. However, this correlation remains under discussion due to unknown variables related to nutrition, customs, and the potential presence of other metal(oid)s. Methods: To determine this association, we sampled water from three Yaqui towns (Cócorit, Vícam, and Pótam), and analyzed the metals by ICP-OES. We exposed four separate groups, with five male CD-1 mice each, to provide further insight into the potential effects of untreated drinking water. Results: The maximum concentrations of each metal(oid) in µg·L^−1^ were Sr(819) > Zn(135) > As(75) > Ba(57) > Mo(56) > Cu(17) > Al(14) > Mn(12) > Se(19). Histological studies revealed brain cells with angulation, satellitosis, and reactive gliosis with significant statistical correlation with Mn and As. Furthermore, the liver cells presented hepatocellular degeneration. Despite the early response, there is no occurrence of both statistical and significative changes in hematological parameters. Conclusions: The obtained results provide experimental insights to understand the potential effects of untreated water with low As and Mn contents in murine models. This fact is noteworthy because of the development of histological changes on both the brain and liver at subchronic exposure.

## 1. Introduction

Among the worldwide populations exposed to toxic metal(oid)s, dispersed rural indigenous communities represent the most disadvantaged group. In arid areas in developing countries, untreated arsenic (As)-contaminated water obtained from wells is the only available drinking water resource [1]. Poor quality of water contributes to environmental health disparities for such minority communities. Arsenic (As) is one of the most important natural pollutants in groundwater used as a drinking water source. Significant As concentrations in groundwater with detectable impacts on human health have been reported in Mexico [2], Vietnam [3], Argentina [4], Chile [5], Nepal [6], Pakistan [7], India [8], Spain [9], Taiwan [10], Thailand [11], and Bangladesh [12].

**Figure 1:**
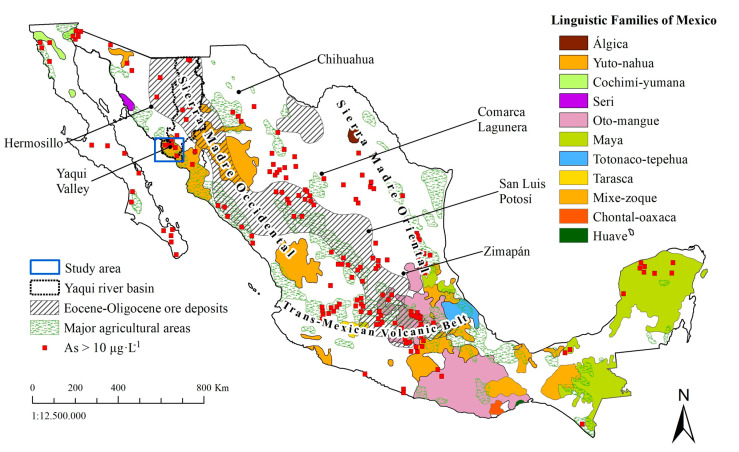
Modified map of Mexico indicating the presence of arsenic (>0.01 mg·L^−1^) in water wells. Location of populations where people speak different language families (indigenous groups), ore deposits, and major agricultural areas are considered. Research in toxicology has been reported for the places indicated with bold lines (e.g., Comarca Lagunera, San Luis Potosí, etc.).

**Figure 2:**
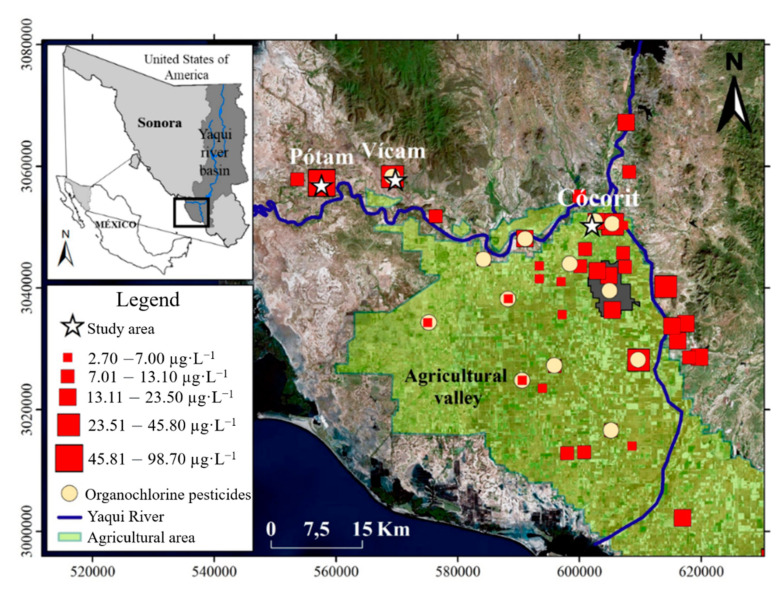
Sample location sites included in the present work. Red boxes indicate arsenic concentrations [24]. Yellow circles represent sampling areas where analysis of organochlorine pesticides have been reported.

The largest indigenous populations in America are concentrated in Mexico and Peru. Because of its toxicological relevance, As is one of the most studied elements in drinking water in Mexico [13]. Figure 1 shows the geographic distribution of linguistic families and the reported As content in water in Mexico. The figure also shows the sites where toxicological studies have been conducted. Most of these works have centered on studying the effects on the population of mining areas located at Chihuahua [14,15], Comarca Lagunera [16], San Luis Potosí [17,18,19], and Zimapán [20]. Still, almost none have focused on investigating vulnerable indigenous communities, where they rarely have water treatment systems. In the Sonora state, northern Mexico, the presence of inorganic arsenic (As) in drinking water has been described above acceptable levels with total As concentrations ranging from 51 to 305 µg·L**^−^**^1^ [21]. Most of the high As-content samples are located within the Yaqui towns [22,23], with 56% of the studied water samples exceeding an As concentration of 10 µg·L**^−^**^1^, the safe limit for drinking water set by both the WHO and the US-EPA [4]. In Mexico, the permissible limit for arsenic in drinking water is 25 µg·L**^−^**^1^ [13]. This study considered the WHO guidelines because they are geared toward human health protection [13]. Lead, copper, cadmium, arsenic, and mercury contents in drinking water from several localities in northern Mexico were reported by Wyatt et al. [21]. Among those metals, As, Pb and Hg were a major concern for some areas of the Sonora state, including As levels above the WHO guidelines in the agricultural Yaqui valley. Other potential pollutants in drinking water include organochlorine pesticides. Figure 2 shows sampling sites where pesticide studies in drinking water have been performed [24]. The reported concentrations for these samples were below detection limits as following: endosulfan < 30 µg·L**^−^**^1^, dichlorodiphenyldichloroethylene (DDE) < 3 µg·L**^−^**^1^, and dichlorodiphenyltrichloroethane (DDT) < 30 µg·L**^−^**^1^.

The Yaqui Valley is the only area in Mexico where toxicological studies have been carried out with indigenous people, and most of these studies are related to exposure to As [22]. The Yaquis are Cahitan-Spanish language speaking indigenous people who inhabit eight towns in the Yaqui River valley in the desertic Sonora region in northern Mexico. In the area, people consume the water obtained from wells without any treatment. Meza et al. determined a correlation between total As in water and urinary excretion of this metalloid [22]. In that study, the percentages of methylated As metabolites were lower than those described in previous works. This finding suggests an individual ability to metabolize and excrete As due to ethnic differences such as the presence of native Indian, Mexican, and Spanish mixture (genetic polymorphism) [22]. Recently, Maldonado-Escalante et al. evaluated the DNA damage (by comet assay) of children from three indigenous towns [25]. The results showed that chronic exposure to the untreated drinking water increased DNA damage, positively correlated with the excreted As in urine. In another study, including 57 more towns located in the same valley, García-Rico et al. reported As content in water and excreted in children’s urine with an assessment indicating that the population is at high risk of developing chronic diseases including cancer [23].

Another study reports a genetic association between polymorphisms of CYT19 and different dimethylarsinic acid (DMAV): monomethylarsonic acid (MMAV) ratios in children, but not in Mexican adults [26]. Children chronically exposed to low-level As in their drinking water produced low% MMAV excreted in their urine. The low% MMAV in urine may protect these children against As-induced toxicity [27]. Some studies have focused on assessing the genotoxic damage of native people from the indigenous towns in the Yaqui Valley. For example, Andrew et al. found that As can affect the repair capacity of DNA by decreasing the expression of the nucleotide excision repair pathway member ERCC1 [28].

Furthermore, an association was reported between increased exposure to As and high concentrations of serum MMP-9 across the native population of the Yaqui Valley. This evidence could indicate that the alteration in MMP-9 is a mechanism that explains the epidemiological link between exposure to As and chronic diseases [29]. Notwithstanding the evidence of biomarkers research showing the exposure and effects of As in populations from the Yaqui Valley, it remains under discussion if there is a direct link to As in drinking water as the main cause of DNA damage in organs such as the brain and liver because of scarce studies. For instance, the As methylation in the Yaqui population has been proved to depend on body mass index, age, and genetics (AS3MT 7388/M287T haplotypes) [30]. Additionally, the inhalation and dermal pathways of human exposure to pesticides and/or As in dust could be present in agricultural areas. Agents such as nutrition [31], habits, sex [32], and sun exposure [33] can influence the toxicity of As. To overcome this issue, the use of animal models has the advantage of analyzing toxic effects under controlled conditions [34,35]. Therefore, the results generated from in vivo experiments can be translated to the toxic effects induced at a biological level in humans [36].

This method is useful for understanding the toxicity of metal(oid)s. Still, in most cases, the provided contaminant is made from synthetic solutions that do not resemble environmental samples**’** complexity, including water [37].

To gain insights into the contribution of As in untreated water to brain and liver damage, mice were used as a model for studying the histological changes induced by untreated drinking water in three indigenous towns.

## 2. Materials and Methods

### 2.1. Study Area

The Yaqui Valley is one of the most important agricultural production areas in Mexico. More than 50% of the basin’s demographics concentrate in this area. The climate of the region goes from dry to extremely dry, with an average annual temperature of 24 °C, the maximum average temperature of 31 °C in the month of June, and the minimum average of 16 °C in January. The Yaqui tribe was established in the coastline of Sonora, but the community was historically displaced, and it is currently living in eight towns within the agricultural Valley (Figure 2). The main economic activity is commercial agriculture. The Yaqui region is independent in politics, military, and religion. Tribal authorities concentrate on the Vícam town. Despite the proximity of the urbanized area of Obregon city, the Yaqui towns lack essential services such as water treatment plants. Water for human consumption is directly extracted from wells and distributed to communities without metal removal treatment.

### 2.2. Water Sample Collection and Analysis

Three water samples of 10 L each were collected directly from wells according to the Mexican Norm (NOM-230-SSA1-2002) [38]. Figure 2 shows sampling sites (Cócorit, Vícam, and Pótam) and the spatial distribution of As in drinking water previously reported [23], as well as the sites where organochlorine pesticides have been analyzed in water with values below the detection limit [24,27]. To collect the water, we used polypropylene bottles that had been previously soaked with detergent, deionized water, and 20% (*v/v*) nitric acid for three days, and finally rinsed with deionized water once again. Samples were packaged on ice and transported in sealed coolers to the laboratory. A portion (500 mL) of each sample was preserved with concentrated nitric acid (pH < 2) before the analysis of total As. The concentration of total metals in the water samples was measured at the University of Sonora with a PerkinElmer 4200 DV ICP-OES coupled with a hydride generation device for As and an ultrasonic nebulizer CETAC UT-5000 for the other elements. The method followed was USEPA 200.7 [39], and the calibration curve was built with four NIST-traceable reference materials (High-Purity Standards, Charleston, SC, USA) and a blank. Three NIST-traceable reference materials (CWW-TM-C, CWW-TM-F, and CWW-TM-G) were used as checking controls with recoveries ranging from 95 to 105%, and the calibration curve coefficient of determination was >0.998 for all analyzed elements. The limit of quantification was 5 µg·L**^−^**^1^ (for Al, Sb, As, Ba, Be, Cd, Cr, Co, Cu, Fe, Pb, Mn, Mo, Ni, Se, Sr, V, and, Zn) and 10 µg·L**^−^**^1^ (for Ag and Tl). The remaining water samples were filtered through a 0.45 µm filter and kept at −4 °C to avoid the transformation of metal(oid)s for the later experiments with mice.

### 2.3. Animals and Treatment

The experiment was carried out following the Official Mexican Standard of the Ministry of Agriculture for the research with laboratory animals titled: “Especificaciones técnicas para la producción, cuidado y uso de los animales de laboratorio” [40]. The ethical committee of the University of Sonora approved all the animal studies, CEI-UNISON 16/2018. Male CD-1 mice, with an average body weight of 24–26 g and 6–8 weeks old, were purchased from the animal facilities of Universidad Autónoma Metropolitana (México). The mice were acclimated to the laboratory environment for seven days before the initiation of treatment. The animals were kept under standard laboratory conditions of temperature (23 ± 2 °C), humidity (50% ± 10%), and a 12 h light/dark cycle. All mice were fed with laboratory rodent diet and water ad libitum. Four separate groups, with 5 mice each, were housed in polypropylene cages with sawdust. We provided each group with water used for human consumption from three different Yaqui populations:

Group I: normal control, animals received Mexican commercial water (Nestlé Waters^®^ Puebla, Mexico) purified and free/low metal(oid)s content.

Group II: mice supplied with drinking water collected from a well in Cócorit;

Group III: mice supplied with drinking water collected from a well in Vícam;

Group IV: mice supplied with drinking water collected from a well in Pótam.

Groups are presented from low to high As and Mn concentrations (see Table 1).

During the experiment, we made daily observations of the general appearance and behavior of the mice. Bodyweight was measured weekly. Animals were sacrificed after 24 weeks by cervical dislocation following the recommended animal euthanasia. For the hematological analysis, blood was drained by cardiac puncture after sacrifice and kept in tubes containing EDTA. The serum was then separated and used for liver function assessment by quantification of serum glutamate-oxaloacetate transaminase (SGOT), serum glutamate-pyruvate transaminase (SGPT), and alkaline phosphatase (ALP). The brains, livers, and other organs were removed and weighed and prepared for further analysis.

### 2.4. Histological Analysis

The brains and livers were dissected and fixed in 10% neutral buffered formalin. Samples were rinsed with 70% ethanol and dehydrated in serial dilutions of ethanol before embedding in paraffin wax. Paraffin blocks of the tissues were sectioned at a thickness of 5 µm. We performed cuts between zone 1–0–1 (coronal section). The outer pyramidal layer, inner granular layer, and inner pyramidal layer were observed. This region of the brain was selected because it is the most affected during an event of As poisoning [41,42]. We processed sections of each block for staining with hematoxylin and eosin (H&E) for histological analysis. In this stain, the nucleic acids appear blue, and the proteins have a red-pink to orange color. This procedure is the standard technique used for the microscopic examination of mouse tissues [43]. Microphotographs of the sections were taken at different magnifications with microscope model Alpha300RA (WITec, Ulm, Germany) using a 50× objective. A pathologist that was blinded to the treatment groups studied the selected fields.

### 2.5. Statistical Analysis

All statistical analyses of blood tissue were done with GraphPad Prism 5. We performed the experiment on 20 mice, with initially *n* = 5 for each treatment. We compared each group the others by applying nonparametric comparison test (Kruskal–Wallis test and Dunn’s test). Data are presented as mean ± SD. Statistical significance was defined as *p* < 0.01.

Principal component analysis (PCA) was performed using the statistical environment R version 3.0.3 (www.R-project.org). For analysis and visualizations, we used the following R packages: ggplot2, dplyr, factomineR, and factoExtra.

## 3. Results

### 3.1. Metal(Oid)s in Drinking Water Used in the Murine Model

The average levels of metal(oid)s in the water samples from the three indigenous towns are displayed in Table 1. The results indicate that Al, Ba, Cu, Fe, Mn, and Se are in concentrations lower than those established in the guidelines for drinking-water quality by the WHO. Furthermore, Mo, Sr, Tl, and Zn were detected, but no worldwide limits have been established. Sb, Be, Cd, Cr, Co, Pb, Ni, Ag, and V were under the detection limit of the equipment. On the other hand, the levels of As in the water of Vícam and Pótam exceeded these recommendations.

### 3.2. The Murine Model

**Figure 3:**
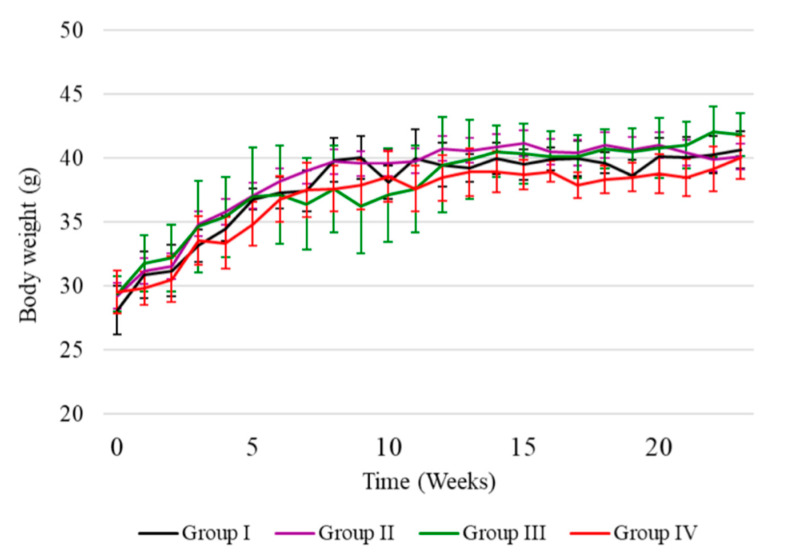
Time evolution of bodyweight of mice exposed to different concentrations of arsenic.

We recorded neither signs of over-toxicity nor death of the mice given the selected untreated water samples used in the experiments. Mice exposed to water from the chosen localities did not present significant changes in body weight concerning the control group (Figure 3). Hence, the mice were well fed, and low concentrations of As and Mn in the water were well tolerated. During subchronic exposure, in routine daily cage checks, we observed incidents of aggression-related injuries. We identify for each group (considering the general appearance and behaviors that may be associated with neurological alterations) the following changes:

Daily routine

Group I (control): No changes in the general appearance, which included the posture, appearance of the coat, nose, eyes, and limbs, were observed.

Group II (exposed to water from Cócorit): We noticed piloerection and stereotypy. These alterations were scarce and occurred when we manipulated the animals for cleaning or measuring body weight.

Group III (exposed to water from Vícam): We noticed neurological changes such as Straub’s sign, piloerection, and stereotypy. The alterations appeared after 18 days of exposure.

Group IV (exposed to water from Pótam): We noticed Straub’s sign, stereotypy, fight attempt, and aggressiveness. This group frequently presented lesions (bite marks on ears, back, and tail). The alterations appeared earlier than group III, after ten days of exposure.

### 3.3. Histological Analysis

#### 3.3.1. Brain Cortex

**Figure 4:**
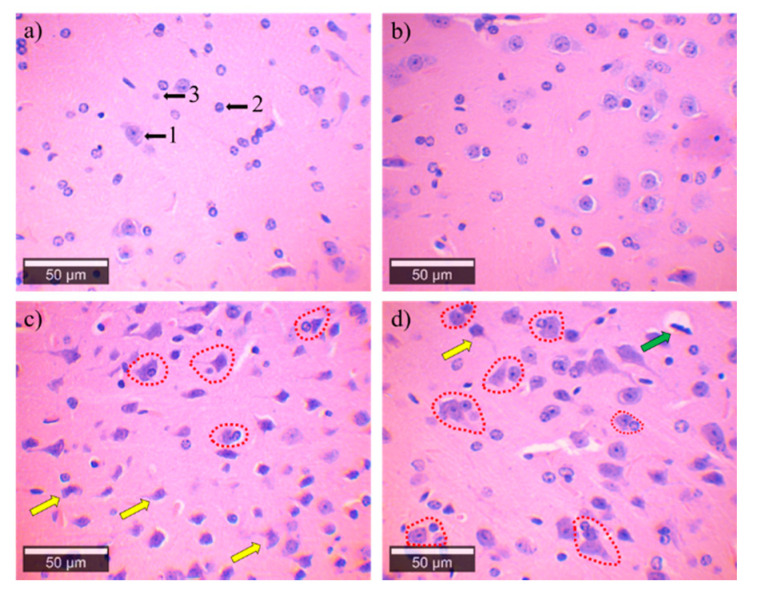
Microphotographs of the brain of the groups exposed to an increase of 50×, 3rd to 4th cortical zone. (**a**) Control group showing the normal structure in the brain cells; the black arrows represent (1) pyramidal neurons, (2) astrocyte and (3) oligodendrocyte. (**b**) Brain cells of the Cócorit group without apparent alterations. (**c**,**d**) Mice brains are exposed to water from Vícam and Pótam, respectively. These groups showed satellitosis (red), neuronal vacuolation (green arrow), and angular degeneration of neurons (yellow arrow).

In our work, hematoxylin and eosin-stained samples of the cerebral cortex of groups I and II showed a healthy structure when observed under an optical microscope (Figure 4a,b). The mouse brain cells from groups III and IV presented degenerative alterations such as angulation, axon retraction, reactive gliosis, neuronal vacuolation, and satellitosis (Figure 4c,d).

#### 3.3.2. Liver

**Figure 5:**
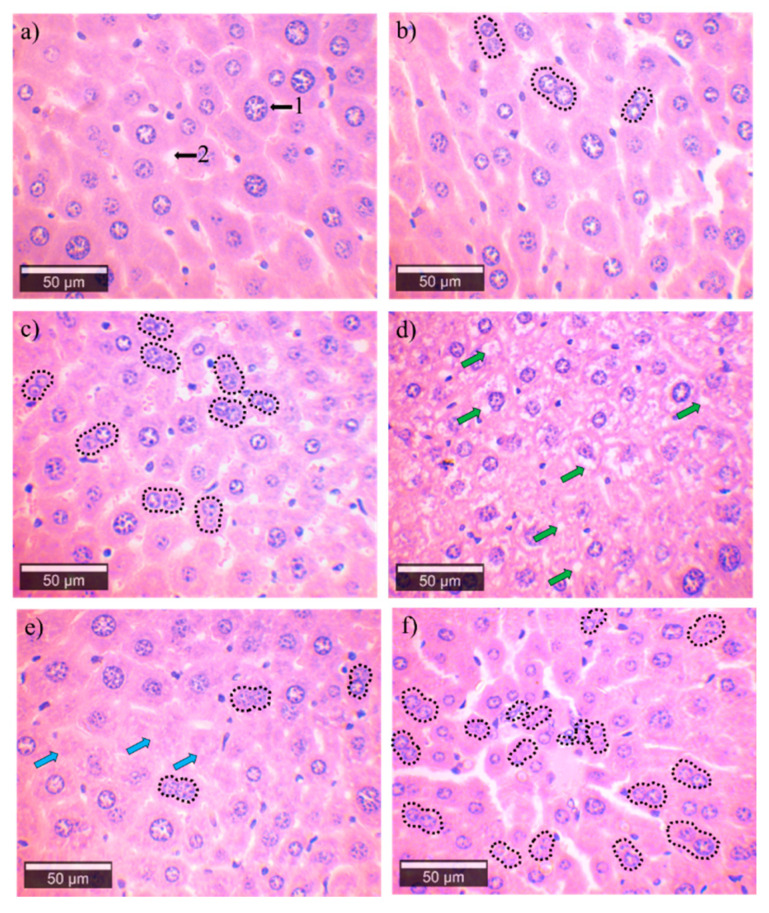
Microphotographs of the medial areas of the mice liver to an increase 50×, (1) sinusoid and (2) hepatocyte. (**a**) Control group showing the normal structure in hepatocytes. (**b**) Cócorit, (**c**) Vícam, and (**d**) Pótam groups show regeneration of hepatocytes (binucleations) (black); and vacuolation (green arrow). Pótam group showing (**e**) necrotic hepatocytes (blue arrows) and (**f**) regenerative binucleation (black).

The histological analysis of the livers of the mice in group I showed no histopathological changes (Figure 5a). The hepatocytes of all the groups exposed to water with As shown a cell injury pattern, characterized by hepatocellular degeneration, vacuolation, and cell death (Figure 5b–d. In all cases, the mice exposed to water with As presented hepatic degeneration. The morphological analysis showed cells with necrotic characteristics (Figure 5e). In addition, we observed a regenerative response characterized by a marked increase in binucleated cells (Figure 5f). All these pathological findings are listed in Table 2.

### 3.4. Hematological and Biochemical Analyses

Although blood is not a good specimen for screening As it can be useful by determining changes in its components that provide information about the effect of drinking water with As. The ingestion of inorganic As is related to a decrease in the production of red and white blood cells. Samples were analyzed in a biochemical laboratory to examine the red and white blood cells, erythrocytes (red blood cells), leukocytes (monocytes, neutrophils, and lymphocytes), and thrombocytes (platelets). Moreover, tests of liver function to determine any injury were carried out. The test panel measured liver enzymes, including two alanine transaminases: SGOT and SGPT, and ALP. These tests may have an equivalent significance to the biomarkers used widely for chronic exposure. In our study, none of the blood tests showed alterations with respect to the control group (Table 3). No significant differences were found among the groups *p* > 0.01.

## 4. Discussion

This work assesses the effects on mice exposed to water with different concentrations of As. The water was obtained from wells of three indigenous populations located in northwestern Mexico. These communities extract water directly from the wells to carry out their daily activities without applying any purification process. In our experiment, the mice exposed to 12 and 75 µg·L*^−^*^1^ showed behavioral alterations. Noteworthy, we observed aggressive behavior in group IV exposed to the highest dose of As-Mn when compared to the control. The potential effects of untreated water on aggression-related injuries should be the subject of future studies. Previous studies have shown alterations in mice’s social behavior resulting from exposure to As [44,45]. Aggressiveness had not been previously reported even at higher doses of the more toxic As III species (NaAsO_2_); instead, it had been discarded [46]. This disagreement could be due to our use of untreated environmental water containing several metal(oid)s, while Moreno Avila et al. used synthetic prepared water with As [46]. Previously, it has been shown that As can pass through the blood–brain barrier and accumulate in the brain [47,48]. Even more, in a similar period in that, we observed behavior alterations, As is distributed in mouse brains [49]. In accordance with this, our study provides experimental information on the damage to brain cells in the two populations with the highest concentration of this metalloid. Chronic As exposure is associated with increased production of reactive oxygen species (ROS) and oxidative stress [50,51]. ROS are the main participants in damage caused by neurodegenerative processes, including cell death [52,53]. Following this, the ROS production triggered by As accumulation in mice brains has been associated with neurobehavioral changes, mainly anxiety and depression [54,55]. Moreover, we detected degenerative changes in cerebral cortex neurons, such as those reported by Selim et al. [56] and alterations in behavior from the first days of exposure that was characterized by a marked aggressiveness in group IV (the mice fed with drinking water containing the highest concentration of As of the three sources sampled).

**Figure 6:**
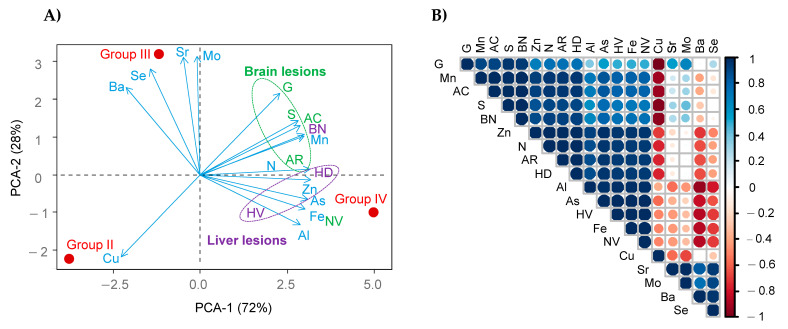
(**A**) Principal component analysis (PCA) loadings of metal(oid)s and liver/brain lesions in Groups II, III and IV. Abbreviations are angulated cells (AC), satellitosis (S), gliosis (G), axon retraction (AR), neuronal vacuolation (NV), hepatocyte vacuolation (HV), hepatocellular degeneration (HD), necrosis (N), binucleated cells (BC). (**B**) Correlation matrix.

Because mice were simultaneously exposed to mixed metals(oid)s in untreated drinking water, a principal component analysis (PCA) was performed to find possible associations between the contaminants and the identified brain and liver lesions. Figure 6 shows the biplot. The first component explains 72% of the total variance. It separates group IV (with the highest levels of As, Mn, and Zn in water) from groups II and III. This principal component has a-positive-loading of Mn strongly associated with identified brain lesions in mice from group IV (gliosis, satellitosis, angulated cells, and axon retraction). Al, As, Fe, and Zn correlated with hepatocyte vacuolation, hepatocellular degeneration (liver lesions), and neuronal vacuolation (brain lesions). Neurotoxic effects of Mn are commonly linked to oxidative stress and neuroinflammation [57].

Additionally, long-term As-exposure may lead to delayed neurodegenerative effects with a progressive loss of neural tissue. It is possible to observe disturbances in motor capacity, intelligence, and psychological effects [31,58].

This fact maybe is related to the high prevalence of child-cancer in the studied region, coming in third place in Mexico. Chronic exposure to As has been reported to be associated with liver damage [59,60]. The diseases and deterioration of the liver that have been described in previous investigations included hepatocellular steatosis, granulomas, cirrhosis fibrosis, and portal hypertension [61,62,63]. The histological analysis of the livers showed that the hepatocytes of all the groups were exposed to water with As shown a cell injury pattern. The effects observed could be associated with the generation of free radical species in the liver [34]. Liver cell necrosis and apoptosis have previously been related to As exposure [64]. In our work, the morphological analysis showed cells with necrotic characteristics, which agrees with previous reports [65,66] where the liver presented a degree of necrosis and degenerative changes in the hepatocytes. In addition, we observed a regenerative response characterized by a marked increase in binucleated cells. In this regard, we observed that although a toxic reaction was identified in the liver, the mice could adapt to the level of exposure to As.

On the contrary, the hematic biometry results, used as the first test of contact with toxic materials, have not reflected a reliable biomarker. The earliest induced effects are analogous to those described in worldwide experiments with high concentrations of As over prolonged or chronic exposure. This fact indicates the importance of studying As in water reservoirs for human consumption, regardless of the low concentration of this metalloid.

## 5. Limitations of the Study

This study shows the following limitations: we did not obtain the physicochemical characteristics of the drinking water. It would have been useful to state a procedure for the isolation of these parameters. The obtained information may account for the toxicity of each contaminant and could provide us with a set of specific contaminants in the area of study. The potential influence of hardness, pH, electric conductivity, and major cations such as Ca^2+^, Na^+^, K^+^, Mg^2+^, and anions such as CO_3_^2−^, NO^3−^, SO_4_^2−^, Cl^−^ remain unknown and require further investigation. Another limitation is that we use a small group of twenty male mice. A group of mice exposed to synthetically prepared water in the laboratory was not included. This restriction is because tap water is an environmental media hard to mimic. In addition, the selected murine model did not resemble human metabolism nor the excretion patterns of As. The study was focused on evaluating histological changes in the brain and liver. However, the observed damage is evident in the exposed Pótam group. This research underlines the necessity of using actual environmental matrices in murine models to provide information to understand toxicological mechanisms.

## 6. Conclusions

It was determined both the presence and concentration of metal(oid)s in tap water from three indigenous towns. The results indicated the all the metal(oid)s were below the safety limit established by WHO, except for As in Vícam and Pótam towns. The concentration of As in this last locality exceeded the mentioned regulations with a value of 75 µg·L*^−^*^1^. The effects of exposure to this As-enriched water containing a complex mix of metals (Sr, Zn, Ba, Mo, Cu, Al, Mn, Se) were observed in mice. The prompt response of the mice to the untreated water shown after ten days consisted of several behavioral changes. Moreover, brain lesions for subchronic As exposure had not been reported until now.

Furthermore, significant histological alterations were observed in the brain and liver. In contrast, the results of hematic biometry, commonly used as the initial test for contact with toxic materials, have not reflected a reliable biomarker. The early induced biological changes are analogous to those described in experiments with high concentrations of As over a long period of chronic exposure. The presence of other metals and contaminants in a synergic combination may generate this amplifying effect. This fact points out the importance of studying complex mixtures of metals in water reservoirs for human consumption, regardless of their low concentrations.

## Figures and Tables

**Table 1:** Concentration of metal(oid)s in drinking water (µg·L^−1^) provided to mice in this work. The indigenous communities from the Yaqui Valley are Pótam, Vícam, and Cócorit. WHO limits taken from Guidelines for drinking-water quality: fourth edition incorporating the first addendum. Geneva: World Health Organization; 2017. License: CC BY-NC-SA 3.0 IGO. R^2^ correlation coefficient of calibration curve (*n* = 5).

Element	R^2^	Cócorit	Vícam	Pótam	WHO Limit
Al	1	13	11	24	100
Sb	0.9999	<DL	<DL	<DL	20
As	0.9984	6	12	75	10
Ba	1	35	57	14	1300
Be	1	<DL	<DL	<DL	-
Cd	1	<DL	<DL	<DL	3
Cr	1	<DL	<DL	<DL	50
Co	1	<DL	<DL	<DL	-
Cu	1	17	7	7	2000
Fe	1	<DL	<DL	19	300
Pb	1	<DL	<DL	<DL	10
Mn	1	<DL	7	12	100
Mo	1	12	56	34	-
Ni	0.9989	<DL	<DL	<DL	70
Se	0.9994	7	10	6	40
Ag	1	<DL	<DL	<DL	-
Sr	1	56	819	121	-
Tl	0.9996	<DL	7	<DL	-
Zn	1	37	62	135	-
V	1	<DL	<DL	<DL	-

**Table 2:** Histopathological grading of liver and brain following exposure to low concentrations of arsenic in water. Here, N = 5. -, nil; +, minimal (<15%); ++, mild (<25%); +++, moderate (<45%) and ++++, severe (>45%).

Lesions	Group I (Control)	Group II (Cócorit)	Group III (Vícam)	Group IV (Pótam)
Brain				
Angulated cells	-	-	++	+++
Satellitosis	-	-	+++	++++
Gliosis	-	-	+++	+++
Axon retraction	-	-	+	++
Neuronal vacuolation	-	-	-	+
Liver				
Hepatocellular degeneration	-	-	+	++
Hepatocyte vacuolation	-	-	-	++
Necrosis	-	-	+	++
Binucleated cells	-	+	+++	++++

N = 5. -, nil; +, minimal (<15%); ++, mild (<25%); +++, moderate (<45%) and ++++, severe (>45%).

**Table 3:** Hematological and biochemical results from sacrificed mice at the end of the experiment. No statistically significant changes were observed.

	Group I (Control)	Group II (Cócorit)	Group III (Vícam)	Group IV (Pótam)
Hematic biometry				
Erythrocytes (×1000 × mm^3^)	7.48 ± 0.54	7.97 ± 0.55	7.71 ± 0.21	7.16 ± 1.91
Platelets (×1000 × mm^3^)	1103.5 ± 698.20	1610.2 ± 257.68	925.5 ± 19.09	803.4 ± 490.52
Leukocytes (×1000 × mm^3^)	7.9 ± 2.29	5.74 ± 4.10	2.70 ± 0.14	6.08 ± 3.53
Monocytes	6.90 ± 6.89%	5.34 ± 4.56%	3.65 ± 2.05%	6.25 ± 2.57%
Neutrophils	11.77 ± 9.76%	9.38 ± 5.43%	11.95 ± 0.64%	6.08 ± 1.37%
Lymphocytes	81.03 ± 16.24%	84.82 ± 4.18%	83.65 ± 1.34%	87.25 ± 3.26%
Hepatic profile				
Serum glutamate-oxaloacetate transaminase (IU*·*L^−1^)	252.50 ± 179.37	214.00 ± 218.27	437.50 ± 340.53	420.00 ± 444.96
Serum glutamate-pyruvate transaminase (IU*·*L^−1^)	55.75 ± 30.24	55.80 ± 52.95	131.25 ± 83.30	90.00 ±87.25
Alkaline phosphatase (IU*·*L^−1^)	40.00 ± 13.54	34.33 ± 10.69	29.50 ± 5.97	23.20 ± 9.52

## Data Availability

The data presented in this study are available in the article.
